# Effects of dimethyl sulfoxide on asymmetric division and cytokinesis in mouse oocytes

**DOI:** 10.1186/1471-213X-14-28

**Published:** 2014-06-21

**Authors:** Dongjie Zhou, Xinghui Shen, Yanli Gu, Na Zhang, Tong Li, Xi Wu, Lei Lei

**Affiliations:** 1 Department of Histology and Embryology, Harbin Medical University, Harbin, China

**Keywords:** Dimethyl sulfoxide (DMSO), Asymmetric division, Actin cap, Spindle migration, Oocyte maturation

## Abstract

**Background:**

Dimethyl sulfoxide (DMSO) is used extensively as a permeable cryoprotectant and is a common solvent utilized for several water-insoluble substances. DMSO has various biological and pharmacological activities; however, the effect of DMSO on mouse oocyte meiotic maturation remains unknown.

**Results:**

In DMSO-treated oocytes, we observed abnormal MII oocytes that contained large polar bodies, including 2-cell–like MII oocytes, during *in vitro* maturation. Oocyte polarization did not occur, due to the absence of actin cap formation and spindle migration. These features are among the primary causes of abnormal symmetric division; however, analysis of the mRNA expression levels of genes related to asymmetric division revealed no significant difference in the expression of these factors between the 3% DMSO-treated group and the control group. After each “blastomere” of the 2-cell–like MII stage oocytes was injected by one sperm head respectively, the oocytes still possessed the ability to extrude the second polar body from each “blastomere” and to begin cleavage. However, MII oocytes with large polar bodies developed to the blastocyst stage after intracytoplasmic sperm injection (ICSI). Furthermore, other permeable cryoprotectants, such as ethylene glycol and glycerol, also caused asymmetric division failure.

**Conclusion:**

Permeable cryoprotectants, such as DMSO, ethylene glycol, and glycerol, affect asymmetric division. DMSO disrupts cytokinesis completion by inhibiting cortical reorganization and polarization. Oocytes that undergo symmetric division maintain the ability to begin cleavage after ICSI.

## Background

Mouse oocyte maturation is a precise and orderly multistage process [1,2]. During this process, the oocyte is transformed into a highly polarized large metaphase II (MII)-arrested oocyte with a small polar body that extrudes from the oocyte, which is essential for the retention of maternal components necessary for early development [3]. Therefore, mouse oocyte meiotic maturation is characterized by a unique asymmetric division process.

To ensure asymmetry in each division, the meiotic spindle has to be positioned near one side of the cortex. During meiosis I (MI), the spindle assembles in the central cytoplasm 2 hours after germinal vesicle breakdown (GVBD) [4]. The spindle migrates along its long axis to the cortex in an actin-dependent manner, which begins approximately 2.5 ± 0.3 hours before polar body extrusion. The spindle does not elongate during anaphase/telophase I, and the first polar body undergoes emission [5]. An actin filament (F-actin)–enriched cortical domain surrounded by a myosin II ring assembles above the meiotic spindle in late MI- to MII-arrested oocytes [6,7]. Assembly of this actomyosin domain is accompanied by microvilli denudation and the exclusion of cortical granules, which redistribute to form a cortical granule-free domain, and the microfilaments are enriched to form an actin cap [8-12]. These changes are referred to as cortical reorganization and polarization, respectively [12-14].

Dimethyl sulfoxide [(CH_3_)_2_SO] is an amphipathic molecule with a highly polar domain and two apolar groups, making it soluble in both aqueous and organic media. Therefore, DMSO is widely used as a solvent for chemicals that have little or no solubility in water [15]. Previous studies have shown that DMSO has various effects on the cell cycle, differentiation, aging, and apoptosis [16,17]. In addition to DMSO, ethylene glycol and other alcohols are frequently used as cryoprotectants, to protect frozen oocytes and embryos for storage purposes and for use as vehicles for drug therapy [17,18]. However, less attention has focused on whether DMSO affects the asymmetric division that occurs in culture media by acting as a solvent for specific growth factors, hormones or meiotic modulators.

Therefore, we investigated the influence of DMSO on asymmetric division during mouse oocyte meiotic maturation.

## Methods

### Animals and reagents

B6D2F1 mice were purchased at 6–8 weeks of age from Vital River Laboratory Animal Technology Co. Ltd (Beijing, China). Mice were maintained in accordance with a light control cycle. This study was conducted in strict accordance with the recommendations in the Guide for the Care and Use of Laboratory Animals. The protocol was approved by the Institutional Research Board of Harbin Medicine University (HMUIRB20140003). Unless otherwise noted, all reagents for embryo culture were purchased from Sigma.

### Oocyte collection and culture

Hybrid B6D2F1 (C57BL/6 × DBA) female mice (6–8 weeks old) were killed 46 hours after administration of pregnant mares serum gonadotrophin (PMSG) in doses of 5 IU. Large follicles on the ovary were ruptured in HEPES to obtain cumulus oocyte complexes (COCs). Cumulus cells were removed by treating the complexes with 0.1% hyaluronidase for 3 minutes. GV oocytes were selected and maintained in CZB medium. Oocytes were covered with sterile mineral oil (Fisher, O121-20) under 5% CO_2_ at 37°C. Oocytes were collected at various time points of culture for immunostaining and real-time RT-PCR analysis.

### DMSO and other cryoprotectant treatment

GV-stage oocytes were collected, washed three times in HEPES, and randomly cultured in the same medium containing 0.5–6% (v/v) DMSO to allow development to the MII stage. Control oocytes were cultured in CZB medium. Oocytes at the GVBD stage (4 hours after GV) were allowed to mature in 2%, 3%, or 4% (v/v) concentrations of DMSO, ethanol, or glycerol to the MII stage.

### Immunofluorescent staining and confocal microscopy

After 8, 9.5, or 12 hours of culturing in CZB or 3% DMSO-CZB medium, the oocytes were collected. Embryos were collected 24 hours after the injection of two sperm heads. The protocol basically followed the methods described in our previous work [19]. In brief, the oocytes were fixed in 4% paraformaldehyde for 40 minutes at room temperature. After permeabilization with 0.3% (v/v) Triton X-100 for 30 minutes, the oocytes were incubated with anti-β-tubulin antibodies (1:100) for 1 hour at 37°C, followed by TRITC–anti-mouse IgG (1:100). Subsequently, the oocytes were incubated in FITC-phalloidin (1:100) for l hour. Nuclei were stained with bisbenzimide Hoechst 33342. All of the samples were mounted on slides with anti-fading medium (DABCO) and examined with a laser-scanning confocal microscope (Zeiss, LSM700). Each experiment was repeated 3 times, and at least 10 samples were observed each time.

### Real-time RT-PCR analysis

Gene expression was measured by real-time RT-PCR and the ΔΔCT method. Oocytes were collected at 12 hours at the MII stage. RNA extraction and quantitative PCR (qPCR) evaluation were performed as previously described, with modifications [20]. Messenger RNA (mRNA) was isolated from each group by using the Dynabeads® mRNA Direct^TM^ Kit (Invitrogen, Cat no.61012) in accordance with the manufacturer's instructions. The cDNA was synthesized from the total RNA with the High-Capacity cDNA Reverse Transcription Kit (ABI, 4368814). Real-time PCR reactions were performed with 1.5 μl of cDNA sample, 10 μl of TransStart^TM^ Top Green qPCR SuperMix (TransGen, mAQ131), and gene-specific primers in a 20-μl reaction system with the CFX96 Real-Time System (Bio-Rad, USA). All of the primers used for the PCR are listed in Table 1.

**Table 1:** Primers used for real-time PCR

**Genes**	**GenBank accession No.**	**Forward**	**Reverse**
*Cdc42*	NM_021454	ATTATGACAGACTACGACCGCT	AGTGGTGAGTTATCTCAGGCA
*Fyn*	NM_008054	ACCTCCATCCCGAACTACAAC	CATAAAGCGCCACAAACAGTG
*Ran*	NP_033417	CCACTTGACGGGCGAGTTT	CCACACAATACAATGGGGATGTT
*Jmy*	NM_021310	AGCAGGAGATCGACGCTCTAT	CCGTCTCGGTGAAAAGAACCT
*Ndc80*	NM_023294	GCCTCTCTATGCAGGAGTTAAGG	CGGTTTGTGTGTACTCAGCTT
*Mos*	NM_020021	CTGTGTCGCTACCTCCCTC	ACCGTGGTAAGTGGCTTTATACA
*Nuf2*	NM_023284	TCCCCAGATACAATGTAGCTGA	CCGGACTCCATACACTAACTGT
*Wave2*	(AY135643	CCGTCAGACGTTGCCTAGC	CCGAAAATGTCCTCTGCATACT
*Arp2/3*	NM_008054	TGAAGCGGACAGGACATTGAT	AGTTGGTGATTCCTAGCGTGT

### Intracytoplasmic sperm injection

ICSI was conducted with a piezo-driven unit following methods previously described elsewhere [21,22], except that our experiment was performed in HEPES-CZB containing 5 μg/ml cytochalasin B at room temperature [19]. One sperm head was injected into each of the two “blastomeres” of the 2-cell–like MII stage oocyte respectively, or one sperm head was injected into the bigger “blastomere” of MII oocytes with large polar bodies. After 10–20 minutes of recovery, the ICSI-generated embryos were washed several times and cultured in KSOM at 37°C in a 5% CO_2_ atmosphere.

### Statistical analysis

All data were obtained from at least three independent experiments. Statistical analysis of the data was performed with Student’s t-test. P < 0.05 was considered statistically significant. All data are shown as mean ± SD.

## Results

### DMSO treatment causes disruption of asymmetric division

**Figure 1:**
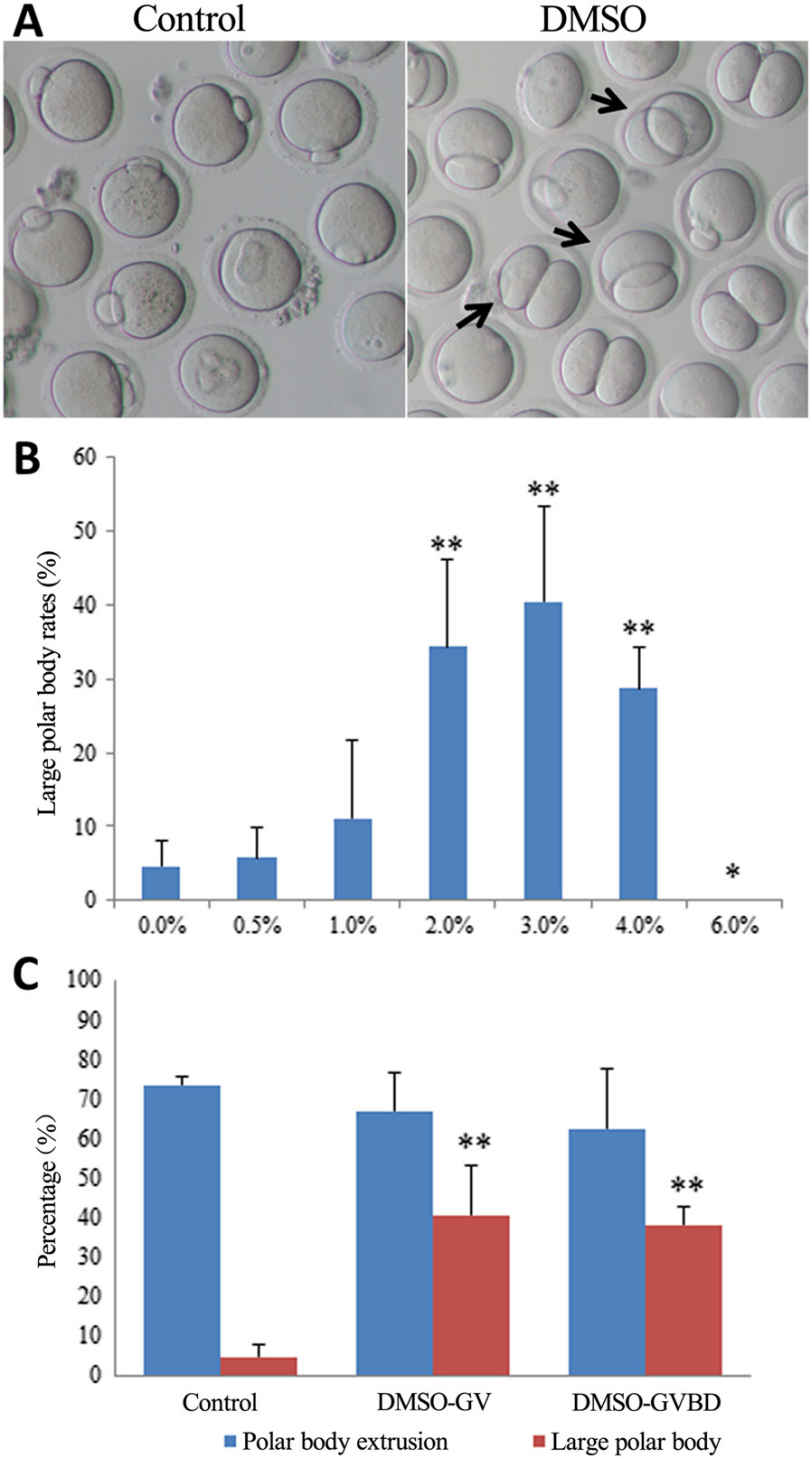
**Effect of DMSO on asymmetric cell division in mouse oocytes. (A)** Abnormal cell division was observed in several DMSO-treated oocytes at the MII stage (arrows). **(B)** Rate of large polar body formation after treatment with various concentrations of DMSO (**p < 0.01, *p < 0.05). **(C)** Rates of PB extrusion and large PB formation when oocytes from GV and GVBD were treated with 3% DMSO (**p < 0.01).

*In vitro*-maturing oocytes were exposed to DMSO, which resulted in abnormal MII oocytes with large polar bodies (e.g., 2-fold the normal polar body size), including 2-cell–like MII oocytes (Figure 1A). When the germinal vesicle (GV) oocytes were cultured in various concentrations of DMSO for 12 h, abnormal cell division was identified in the MII oocytes treated with 2% to 4% DMSO; however, oocyte maturation was not affected by 0.5% DMSO. Oocytes treated with 6% DMSO failed to mature (Figure 1B). No significant difference in the percentage of oocytes with polar body extrusion or the number of MII oocytes with large polar bodies was observed when the GV and GVBD oocytes were cultured in 3% DMSO (Figure 1C).

### DMSO treatment disrupts chromosome congression, spindle migration, positioning, and actin cap formation

**Figure 2:**
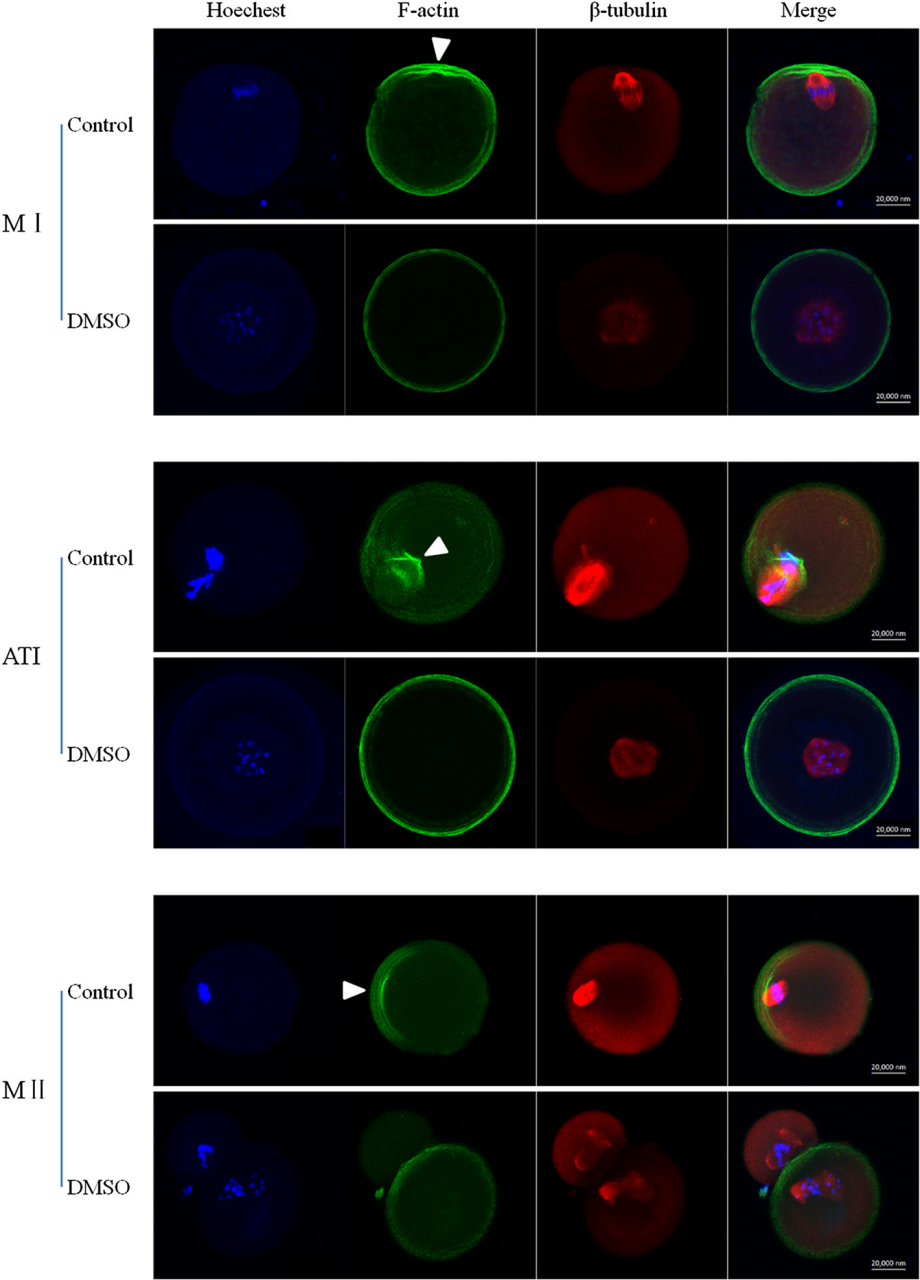
**Immunofluorescent localization of microfilaments and microtubules in mouse oocytes with and without DMSO treatment.** Arrowheads indicate F-actin caps. Green, microfilaments; red, microtubules; blue, DNA. Bar = 20 μm. Representative images from three replicates are shown.

In mammalian oocytes, spindle migration is driven by actin. Using confocal microscopy, we examined the spindle and actin morphologies of oocytes after 8, 9.5, and 12 hours of *in vitro* maturation, representing the time points at which most oocytes reach the MI, anaphase/telophase I (ATI), and MII stages, respectively. In the control group, by the MI stage after 8 hours of culturing, the chromosomes had already moved to the cortex, and the actin cap had formed. At the ATI stage, the chromosomes dissociated at the region of the cortex with the actin cap. By the MII stage, a small polar body and a large MII oocyte had formed (Figure 2).In contrast, in the DMSO group, chromosomes did not move from the center of the cytoplasm, and actin was observed throughout the entire cortex at the MI stage. At the ATI stage, chromosomes remained in the center of the cytoplasm with an abnormal spindle. By the MII stage, several oocytes had formed a 2-cell–like structure (Figure 2). These results indicate that spindle migration and actin cap formation were disrupted after DMSO treatment.

### DMSO has no effect on the expression of the molecular factors that regulate asymmetry division in mouse oocytes

**Figure 3:**
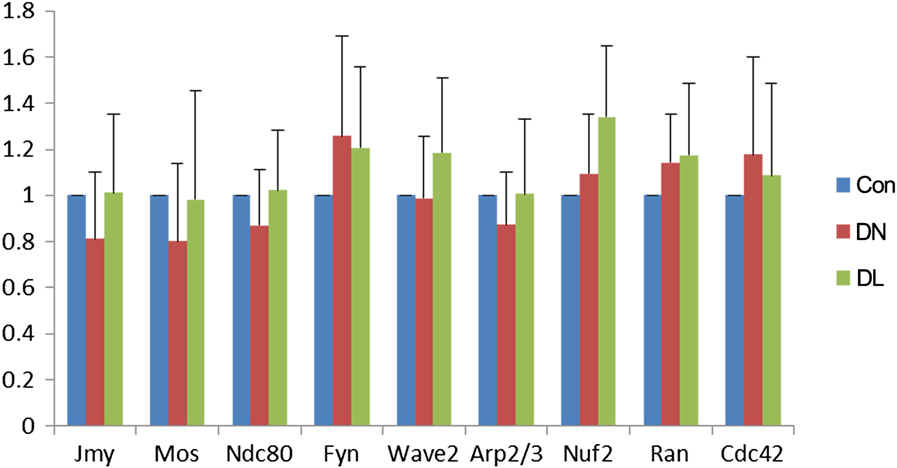
**Effect of DMSO on the expression levels of genes involved in asymmetric cell division.** Expression levels of *Jmy, Mos, Ndc80, Fyn, Wave2, Arp2/3, Nuf2, Ran,* and *Cdc42* were not significantly different between the control group and DMSO-treated group (*p* > 0.05). Con, control group; DN, normal polar body in DMSO group; DL, large polar body in DMSO group.

We further investigated the roles of DMSO during mouse oocyte meiotic maturation by examining the expression levels of crucial regulatory genes involved in asymmetric cell division. No significant differences in the gene expression levels of *Arp2/3, Jmy, Wave2, Mos, Ran, Cdc42, Fyn, Nuf2*, and *Ndc80* were observed between the 3% DMSO-treated group and the control group (Figure 3). Therefore, DMSO does not cause asymmetric division failure by regulating the molecular factors that mediate asymmetry.

### MII oocytes with large polar bodies undergo pre-implantation development after ICSI

**Figure 4:**
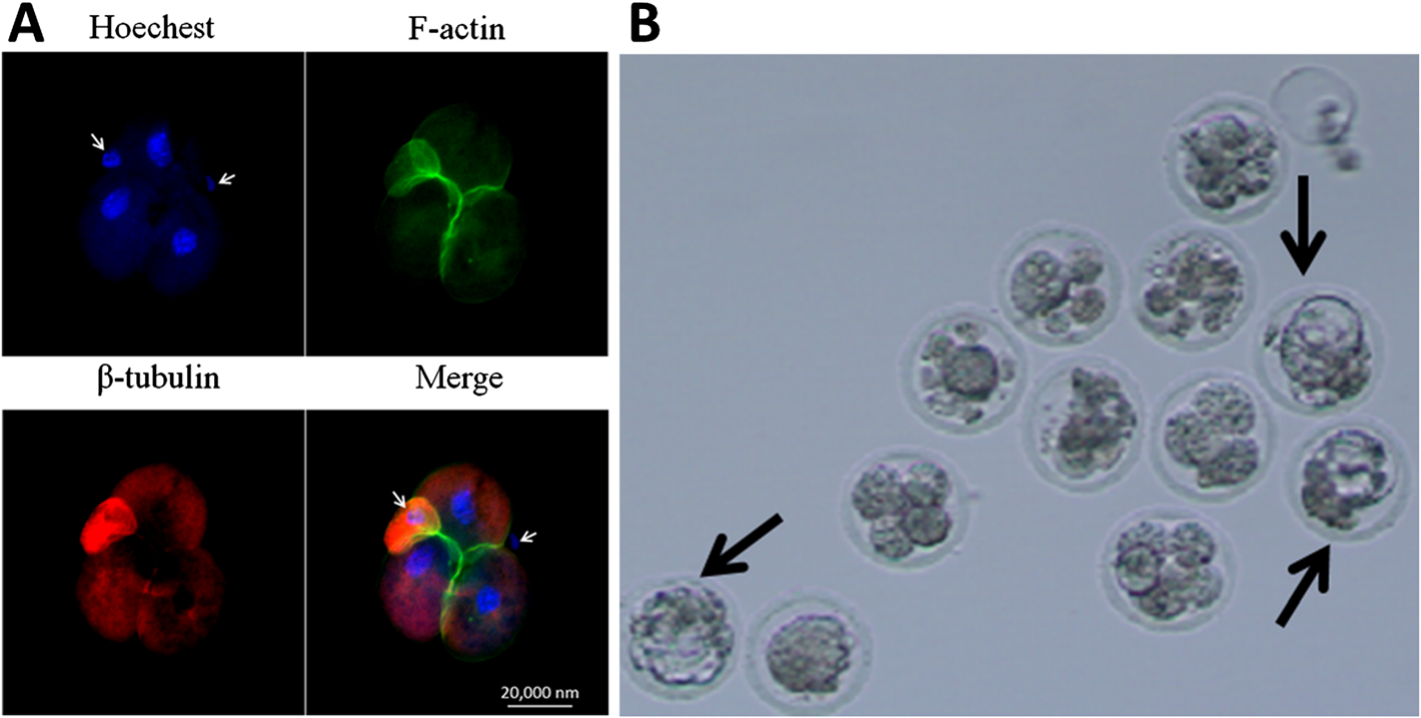
**Pre-implantation development of the ICSI embryos. (A)** Samples were collected after two sperm heads were injected into the 2-cell–like MII oocytes for 24 hours. Time points when most embryos reached the 2-cell stage are shown. Arrowheads indicate the second polar bodies. Green, microfilaments; red, microtubules; blue, DNA. Bar = 20 μm. **(B)** ICSI blastocyst that forms a MII oocyte with a large polar body (arrows).

We next wondered whether MII oocytes with large polar bodies are capable of undergoing pre-implantation development after ICSI. One sperm head was injected into each of the two “blastomeres” of the 2-cell–like MII stage oocytes respectively. After 24 h of ICSI, the second polar bodies were extruded from each of the “blastomeres,” and the embryos had begun cleavage (Figure 4A). Although no blastocyst was observed, the polar bodies of the 2-cell–like MII oocyte also underwent pre-implantation development. When one sperm head was injected into the larger “blastomere” of the MII oocytes with large polar bodies, several ICSI embryos reached the blastocyst stage (Figure 4B). These results indicate that the MII oocytes with large polar bodies that are treated with DMSO undergo pre-implantation development after ICSI.

### Ethylene glycol and glycerol also cause asymmetric division failure

**Figure 5:**
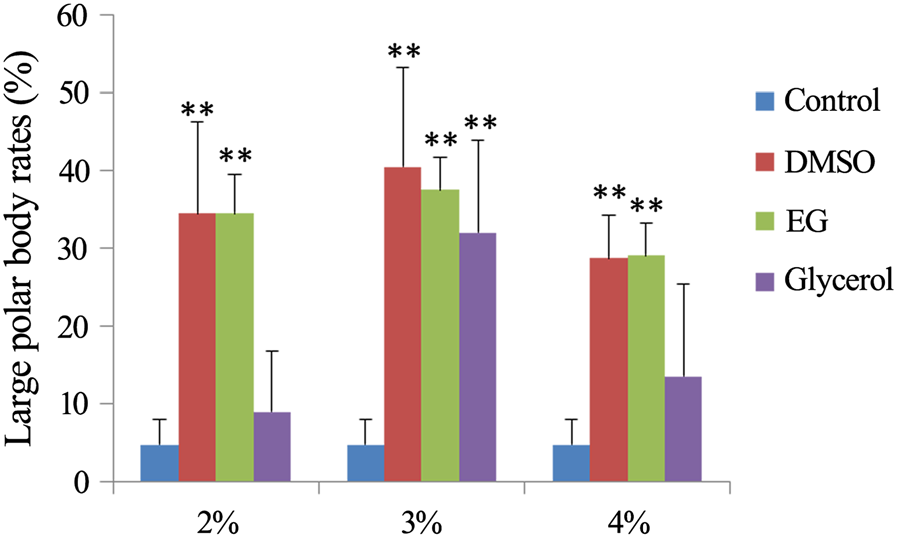
**Effect of cryoprotectants on the asymmetric cell division of mouse oocytes.** Rate of large polar body extrusion after treatment with various concentrations of DMSO, ethylene glycol, or glycerol. EG, ethylene glycol. (**p < 0.01).

DMSO, similar to ethylene glycol and glycerol, exhibits high permeability. GVBD oocytes were cultured in medium containing 2%, 3%, or 4% ethylene glycol or glycerol. MII oocytes with large polar bodies were observed after treatment with 2%, 3%, and 4% ethylene glycol and 3% glycerol (Figure 5). Thus, ethylene glycol and glycerol treatments also cause asymmetric division failure.

## Discussion

The experiments presented above demonstrate that DMSO, ethanol and glycerol have profound effects on the *in vitro* maturation of the mouse oocyte. Small concentrations (≤0.5%) of DMSO had no detectable effect on asymmetric division. Similarly, DMSO had no influence on asymmetric division as a solvent present in the control group medium [23,24]. However, after treatment with a high concentration of DMSO (2–4%), actin distribution was perturbed, and spindle migration, asymmetric division, and cytokinesis completion were disrupted during oocyte meiotic maturation. In addition, treatment with DMSO at concentrations above 6% blocked oocyte maturation. Although the direct targets of DMSO were not identified, this work is the first report describing the effects of DMSO on asymmetric division during oocyte maturation.

Multiple genes related to oocyte polarity, spindle migration, and actin polymerization are involved in mouse oocyte maturation. The overexpression or depletion of genes such as *Arp2/3, Jmy, Wave2, Mos, Ran, Cdc42, Fyn, Nuf2*, and *Ndc80* leads to symmetric cell division [7,24-33]. However, in our study, no significant changes were observed in the mRNA expression levels of these genes between the control group and the DMSO-treated group, suggesting that the disrupted asymmetric division of oocyte meiotic maturation by DMSO is not caused by changes in the mRNA expression of these genes.

Asymmetric oocyte division depends on the position of the spindle that is formed after GVBD. Spindle migration is primarily regulated by actin filaments during oocyte maturation [5,10,34,35]. The cortical actin meshwork was disrupted by the exposure of MII oocytes to 1.5 M DMSO [36]. Actin polymerization is a key regulator of asymmetric oocyte division. We also found that during mouse oocyte maturation, treatment with 1% to 4% DMSO disrupted the polymerization of actin, suggesting that DMSO may disrupt oocyte asymmetric division through the actin meshwork. In addition, a dynamic actin network drives cytoplasmic streaming in a pattern that produces a pushing force on the spindle towards the cortex [35]. Potentially, DMSO disrupts oocyte asymmetric division through actin-driven cytoplasmic streaming. In DMSO-treated oocytes, the cortical granule free domain did not form (data not shown). Taken together, the results demonstrate that DMSO may influence asymmetric division by acting on oocyte polarization.

After the injection of one sperm head into each of the two “blastomeres” of 2-cell–like MII stage oocytes respectively, the oocytes still extruded the second polar body from each “blastomere” and began cleavage; however, no blastocyst was observed. Although MII oocytes with large polar bodies underwent pre-implantation development after ICSI, the blastocyst rate was significantly lower than in the control group. This result is most likely because DMSO disrupts cytokinesis, or the maternal components of the oocyte are not sufficient to support pre-implantation development. Determining the effect of symmetric division during oocyte maturation on post-implantation development will require further research.

DMSO, ethylene glycol, and glycerol are used as cryoprotectants because of their high permeability. In our studies, we found that ethylene glycol and glycerol treatment also caused symmetric division. Thus, the high permeability of these compounds is the likely reason that they cause asymmetric division failure.

## Conclusion

DMSO, ethylene glycol, and glycerol act as permeable cryoprotectants that affect asymmetric division. DMSO disrupts cytokinesis completion by inhibiting cortical reorganization and polarization. In addition, the oocytes that undergo symmetric division still maintain the ability to complete pre-implantation development after ICSI.

## Competing interests

The authors declare that they have no competing interest.

## Authors’ contributions

DZ and XS conceived the study and participated in its design, and drafted the manuscript. YG performed qPCRs, gave advice on the statistical and cartographic. NZ helped in preparing the experiments. TL and WX participated in embryo micromanipulation. LL participated in the design of the study and draft the manuscript. All authors read and approved the final manuscript.

## References

[B1] Niikura Y Niikura T Tilly JL Aged mouse ovaries possess rare premeiotic germ cells that can generate oocytes following transplantation into a young host environment Aging (Albany NY) 2009 1 971 978 20157580 10.18632/aging.100105 PMC2815754

[B2] Niikura Y Niikura T Wang N Satirapod C Tilly JL Systemic signals in aged males exert potent rejuvenat -ing effects on the ovarian follicle reserve in mammalian females Aging (Albany NY) 2010 2 999 1003 21212462 10.18632/aging.100255 PMC3034188

[B3] Maro B Verlhac MH Polar body formation: new rules for asymmetric divisions Nat Cell Biol 2002 4 E281 E283 12461532 10.1038/ncb1202-e281

[B4] Verlhac MH Kubiak JZ Clarke HJ Maro B Microtubule and chromatin behavior follow MAP kinase activity but not MPF activity during meiosis in mouse oocytes Development 1994 120 1017 1025 7600950 10.1242/dev.120.4.1017

[B5] Verlhac MH Lefebvre C Guillaud P Rassinier P Maro B Asymmetric division in mouse oocytes: with or without Mos Curr Biol 2000 10 1303 1306 11069114 10.1016/s0960-9822(00)00753-3

[B6] Brunet S Maro B Cytoskeleton and cell cycle control during meiotic maturation of the mouse oocyte: integrating time and space Reproduction 2005 130 6 801 811 16322540 10.1530/rep.1.00364

[B7] Deng M Suraneni P Schultz RM Li R The Ran GTPase mediates chromatin signaling to control cortical polarity during polar body extrusion in mouse oocytes Dev Cell 2007 12 2 301 308 17276346 10.1016/j.devcel.2006.11.008

[B8] Johnson MH Eager D Muggleton-Harris A Grave HM Mosaicism in organisation concanavalin A receptors on surface membrane of mouse egg Nature 1975 257 5524 321 322 1172195 10.1038/257321a0

[B9] Maro B Johnson MH Pickering SJ Flach G Changes in actin distribution during fertilization of the mouse egg J Embryol Exp Morphol 1984 81 211 237 6540795

[B10] Maro B Johnson MH Webb M Flach G Mechanism of polar body formation in the mouse oocyte: an interaction between the chromosomes, the cytoskeleton and the plasma membrane J Embr yol Exp Morphol 1986 92 11 32 3723057

[B11] Deng M Williams CJ Schultz RM Role of MAP kinase and myosin light chain kinase in chromosome-induced development of mouse egg polarity Dev Biol 2005 278 2 358 366 15680356 10.1016/j.ydbio.2004.11.013

[B12] Longo FJ Chen DY Development of cortical polarity in mouse eggs: involvement of the meiotic apparatus Dev Biol 1985 107 2 382 394 4038667 10.1016/0012-1606(85)90320-3

[B13] Van Blerkom J Bell H Regulation of development in the fully grown mouse oocyte: chromosome-mediated temporal and spatial differentiation of the cytoplasm and plasma membrane J Embryol Exp Morphol 1986 93 213 238 3734683

[B14] Vincent C Pickering SJ Johnson MH Quick SJ Dimethylsulphoxide affects the organisation of microfilaments in the mouse oocyte Mol Reprod Dev 1990 26 227 235 2375876 10.1002/mrd.1080260306

[B15] Santos NC Prieto MJE Morna-Gomes A Betbeder D Castanho MARB Structural characterization (shape and dimensions) and stability of polysaccharide/lipid nanoparticles Biopolymers 1997 41 511 520

[B16] Choi T Dimethyl sulfoxide inhibits spontaneous oocyte fragmentation and delays inactivation of maturation promoting factor (MPF) during the prolonged culture of ovulated murine oocytes in vitro Cytotechnology 2011 63 279 284 21336963 10.1007/s10616-011-9339-8 PMC3081052

[B17] Santos NC Figueira-Coelho J Martins-Silva J Saldanha C Multidisciplinary utilization of dimethyl sulfoxide: pharmacological, cellular, and molecular aspects Biochem Pharmacol 2003 65 1035 1041 12663039 10.1016/s0006-2952(03)00002-9

[B18] Jacob SW Torre JCDL Pharmacology of dimethyl sulfoxide in cardiac and CNS damage Pharmacol Rep 2009 61 225 235 19443933 10.1016/s1734-1140(09)70026-x

[B19] Hu LL Shen XH Zheng Z Wang ZD Liu ZH Jin LH Lei L Cytochalasin B treatment of mouse oocytes during intracytoplasmic sperm injection (ICSI) increases embryo survival without impairment of development Zygote 2011 15 1 9 10.1017/S0967199411000438 21838963

[B20] Suzuki T Minami N Kono T Imai H Comparison of the RNA polymerase I-, II- and III-dependent transcript levels between nuclear transfer and in vitro fertilized embryos at the blastocyst stage J Reprod Dev 2007 53 663 671 17380042 10.1262/jrd.19014

[B21] Tesarik J Rienzi L Ubaldi F Mendoza C Greco E Use of a modified intracytoplasmic sperm injection technique to overcome sperm-borne and oocyte-borne oocyte activation failures Ferti Steril 2002 78 619 624 10.1016/s0015-0282(02)03291-0 12215343

[B22] Araki Y Yoshizawa M Abe H Murase Y Araki Y Use of mouse oocytes to evaluate the ability of human sperm to activate oocytes after failure of activation by intracytoplasmic sperm injection Zygote 2004 12 111 116 15460105 10.1017/s0967199404002606

[B23] Lee SE Sun SC Choi HY Uhm SJ Kim NH mTOR is required for asymmetric division through small GTPases in mouse oocytes Mol Reprod Dev 2012 79 356 366 22407942 10.1002/mrd.22035

[B24] Sun SC Wang ZB Xu YN Lee SE Cui XS Kim NH Arp2/3 complex regulates asymmetric division and cytokinesis in mouse oocytes PLoS One 2011 6 4 e18392 21494665 10.1371/journal.pone.0018392 PMC3072972

[B25] Zuchero JB Coutts AS Quinlan ME Thangue NB Mullins RD p53-cofactor JMY is a multifunctional actin nucleation factor Nat Cell Biol 2009 11 451 459 19287377 10.1038/ncb1852 PMC2763628

[B26] Yamazaki D Suetsugu S Miki H Kataoka Y Nishikawa S Fujiwara T Yoshida N Takenawa T WAVE2 is required for directed cell migration and cardiovascular development Nature 2003 424 452 456 12879075 10.1038/nature01770

[B27] Yan C Martinez-Quiles N Eden S Shibata T Takeshima F Shinkura R Fujiwara Y Bronson R Snapper SB Kirschner MW Geha R Rosen FS Alt FW WAVE2 deficiency reveals distinct roles in embryogenesis and Rac-mediated actin-based motility Embo J 2003 22 3602 3612 12853475 10.1093/emboj/cdg350 PMC165620

[B28] Choi T Fukasawa K Zhou R Tessarollo L Borror K Resau J Vande Woude GF The Mos/mitogen-activated protein kinase (MAPK) pathway regulates the size and degradation of the first polar body in maturing mouse oocytes Proc Natl Acad Sci U S A 1996 93 7032 7035 8692939 10.1073/pnas.93.14.7032 PMC38930

[B29] Na J Zernicka-Goetz M Asymmetric positioning and organization of the meiotic spindle of mouse oocytes requires CDC42 function Curr Biol 2006 16 1249 1254 16782018 10.1016/j.cub.2006.05.023

[B30] Luo J McGinnis LK Kinsey WH Fyn kinase activity is required for normal organization and functional polarity of the mouse oocyte cortex Mol Reprod Dev 2009 76 819 831 19363790 10.1002/mrd.21034 PMC4001720

[B31] McGinnis LK Kinsey WH Albertini DF Functions of Fyn kinase in the completion of meiosis in mouse oocytes Dev Biol 2009 327 280 287 19118543 10.1016/j.ydbio.2008.11.038 PMC2649971

[B32] Meraldi P Draviam VM Sorger PK Timing and checkpoints in the regulation of mitotic progression Dev Cell 2004 7 45 60 15239953 10.1016/j.devcel.2004.06.006

[B33] Sun SC Zhang DX Lee SE Xu YN Kim NH Ndc80 regulates meiotic spindle organization, chromosome alignment, and cell cycle progression in mouse oocytes PLoS One 2011 6 4 e18392 21600073 10.1017/S1431927611000274

[B34] Leader B Lim H Carabatsos MJ Harrington A Ecsedy J Pellman D Maas R Leder P Formin-2, polyploidy, hypofertility and positioning of the meiotic spindle in mouse oocytes Nat Cell Biol 2002 4 12 921 928 12447394 10.1038/ncb880

[B35] Yi K Unruh JR Deng M Slaughter BD Rubinstein B Li R Dynamic maintenance of asymmetric meiotic spindle position through Arp2/3-complex-driven cytoplasmic streaming in mouse oocytes Nat Cell Biol 2011 13 10 1252 1258 21874009 10.1038/ncb2320 PMC3523671

[B36] Deng M Kishikawa H Yanagimachi R Kopf GS Schultz RM Williams CJ Chromatin-mediated cortical granule redistribution is responsible for the formation of the cortical granule-free domain in mouse eggs Dev Biol 2003 257 166 176 12710965 10.1016/s0012-1606(03)00045-9

